# Setback Distances as a Conservation Tool in Wildlife-Human Interactions: Testing Their Efficacy for Birds Affected by Vehicles on Open-Coast Sandy Beaches

**DOI:** 10.1371/journal.pone.0071200

**Published:** 2013-09-05

**Authors:** Thomas A. Schlacher, Michael A. Weston, David Lynn, Rod M. Connolly

**Affiliations:** 1 Faculty of Science, University of the Sunshine Coast, Queensland, Australia; 2 Centre for Integrative Ecology, Deakin University, Burwood, Australia; 3 Australian Rivers Institute – Coast & Estuaries, Griffith University, Gold Coast, Australia; University of Western Ontario, Canada

## Abstract

In some wilderness areas, wildlife encounter vehicles disrupt their behaviour and habitat use. Changing driver behaviour has been proposed where bans on vehicle use are politically unpalatable, but the efficacy of vehicle setbacks and reduced speeds remains largely untested. We characterised bird-vehicle encounters in terms of driver behaviour and the disturbance caused to birds, and tested whether spatial buffers or lower speeds reduced bird escape responses on open beaches. Focal observations showed that: i) most drivers did not create sizeable buffers between their vehicles and birds; ii) bird disturbance was frequent; and iii) predictors of probability of flushing (escape) were setback distance and vehicle type (buses flushed birds at higher rates than cars). Experiments demonstrated that substantial reductions in bird escape responses required buffers to be wide (> 25 m) and vehicle speeds to be slow (< 30 km h^-1^). Setback distances can reduce impacts on wildlife, provided that they are carefully designed and derived from empirical evidence. No speed or distance combination we tested, however, eliminated bird responses. Thus, while buffers reduce response rates, they are likely to be much less effective than vehicle-free zones (i.e. beach closures), and rely on changes to current driver behaviour.

## Introduction

Protected areas generally have a dual mandate of protecting important and irreplaceable features of the natural environment (‘conservation’), whilst providing visitors with opportunities to experience nature or engage in recreational activities [1,2]. Meeting these dual objectives is often difficult, and the ratio between environmental costs and economic benefits is a dynamic one, often driven by political and cultural forces [3]. For example, although the values of conservation in parks can, arguably, outweigh those of tourism and recreation, visitors also create political capital that influences a government’s budget allocations to conservation on public lands [1]. The interactions between wildlife and vehicles are one of the central issues and conservation challenges associated with these twin objectives. Vehicles are a well-documented threat to wildlife through mortality, disturbance (behavioural or physiological disruption), and other processes such as air pollution and habitat alterations [4–6].

The potential for conflict between conservation and human use is starkly evident on sandy beaches and coastal dunes, where motorised transport is a widely practiced recreational activity, especially in Australia and the US. The activity is, however, seldom compatible with the conservation function of beaches and dunes, including the protection of wildlife and habitats [7]. Beaches and dunes are particularly attractive as sites for recreation, but also form unique habitats that contain species and assemblages vulnerable to vehicles: this juxtaposition of spatially concentrated, and often intense, motorised recreation with bio-diverse and malleable habitats results in magnified vehicle effects along many sandy shorelines [8,9]. Impacts of vehicles driven on beaches and dunes are reported from all levels of ecological organisation, including substantial habitat modifications [10–12], significant reductions in biodiversity and lower abundance of plants and invertebrates [13–15], and mortalities or significant disturbance of vertebrates [16,17]. Other processes demonstrated in terrestrial settings, such as the transportation of weeds, also presumably apply to beaches and dunes [18].

In terrestrial environments, roads can act as barriers to wildlife and tend to fragment wildlife habitat. Some beaches are functionally - and sometimes legally - ‘roads’, where the ‘road’ occurs within a large portion of, or encompasses, the habitat of beach fauna [19,20]. Additionally, unlike terrestrial roads, beaches as roads may not host traffic during high water periods (when sand is soft and beaches impassable), and so vehicles may be episodic; unpredictable stimuli that may be more disturbing than predicable benign stimuli [21]. Like other vertebrates, beach-dwelling birds are prone to disturbance when they encounter potentially threatening stimuli, such as humans engaged in recreational activities [21]. On beaches, vehicles also potentially crush eggs and young and cause fatal collisions with birds [22,23]. Lowered reproductive success and adult survival are key demographic parameters which potentially influence population viability, thus the impacts of vehicles potentially represent conservation threats to populations of beach-dwelling fauna.

Bird escape responses are considered anti-predator in nature, regardless of the agent which evokes a response [24]. Escape behaviour balances the risk of staying (risk of collision and death) with the costs of leaving (increased energy expenditure and displacement from habitat) [25]. Although bird responses to human stimuli vary considerably between and within species, two general principles have emerged which can explain a sizeable part of this variation: 1) the probability of flight, or the intensity of the response, decreases with increasing distance between the birds and the stimulus, and 2) attributes of the stimulus (e.g. size and speed) can be important in mediating the responses [24].

In conservation practices, these principles translate into management interventions where establishing separation distances (‘buffers’ or ‘setbacks’) and lowering vehicle speed are thought to reduce the occurrence of collisions and costly escape responses [26,27]. This approach may be particularly important on open-coast beaches, where birds settle in generally unpredictable locations, making engineering solutions to reduce vehicle impacts (e.g. fences, localised signage, small-scale beach closures) impractical [28,29]. Birds get killed in collisions with vehicles when escape behaviours are late or inadequate. Because escape responses most probably evolved in relation to the speed of natural predators rather than modern motor cars, lowering vehicle speed limits may permit birds to better respond to vehicles.

Because setback distances differ greatly between species and depend on the environmental and biological context [30], their applicability as a conservation tool to specific situations needs to be supported by empirical evidence. Because all setback distances require adoption and compliance by humans interacting with wildlife [27], evidence of their efficacy should improve uptake and, ultimately, conservation effectiveness. In this context, there were three key information gaps which we addressed in this study: 1) essential attributes defining vehicle-wildlife interactions on beaches are ill-defined, 2) the metrics for setback distances during vehicle encounters have not been determined, and 3) the efficacy of setback distances is undocumented for open-coast beaches.

Significant conservation concerns for coastal birds exist [31], some of which could, hypothetically, be ameliorated by the adoption of - largely untested - setback tools to reduce the impacts from vehicle encounters on open beaches [26]. We used both observational and experimental approaches to address four inter-related objectives: i) to describe the nature of encounters between motorised recreationists and birds on open-coast beaches (‘driver behaviour’), ii) to quantify the frequency and intensity of disturbance responses by birds resulting from these encounters (‘disturbance effects’), iii) to identify factors that influence the probability of birds escaping by flight (‘determinants of probability of flushing’), and iv) to test the efficacy of alternative setback distances and encounter speeds to reduce the probability of flushing (‘tool evaluation’). Our model system were the open-coast beaches of two barrier islands in Eastern Australia where interactions between birds and vehicles are frequent, leading to conservation concerns about the practice of beach driving and its management.

## Materials and Methods

### Ethics statement

All beach sites are open to members of the public and open to vehicles with a permit. Vehicle access permits were purchased through standard channels used by the general public / recreational users (e.g. http://www.straddiecamping.com.au/4wds.php#4wd-permits). Observations on disturbance of birds by other beach users did not involve direct contact with vertebrates or the beach users nor did the experiments. The latter were no different than the thousands of encounters that birds experience from vehicles used by the general public on these beaches. Neither of the two bird species in the experiments is currently listed as endangered or protected http://www.ehp.qld.gov.au/wildlife/threatened-species/endangered/endangered-animals/index.html#birds_16_species). No vertebrates were collected, sampled, sacrificed, or physically harmed in any way. The work was carried out under animal ethics permit AN/A/10/56, issued by the Animal Ethics Committee of the University of the Sunshine Coast.

### Study area

**Figure 1:**
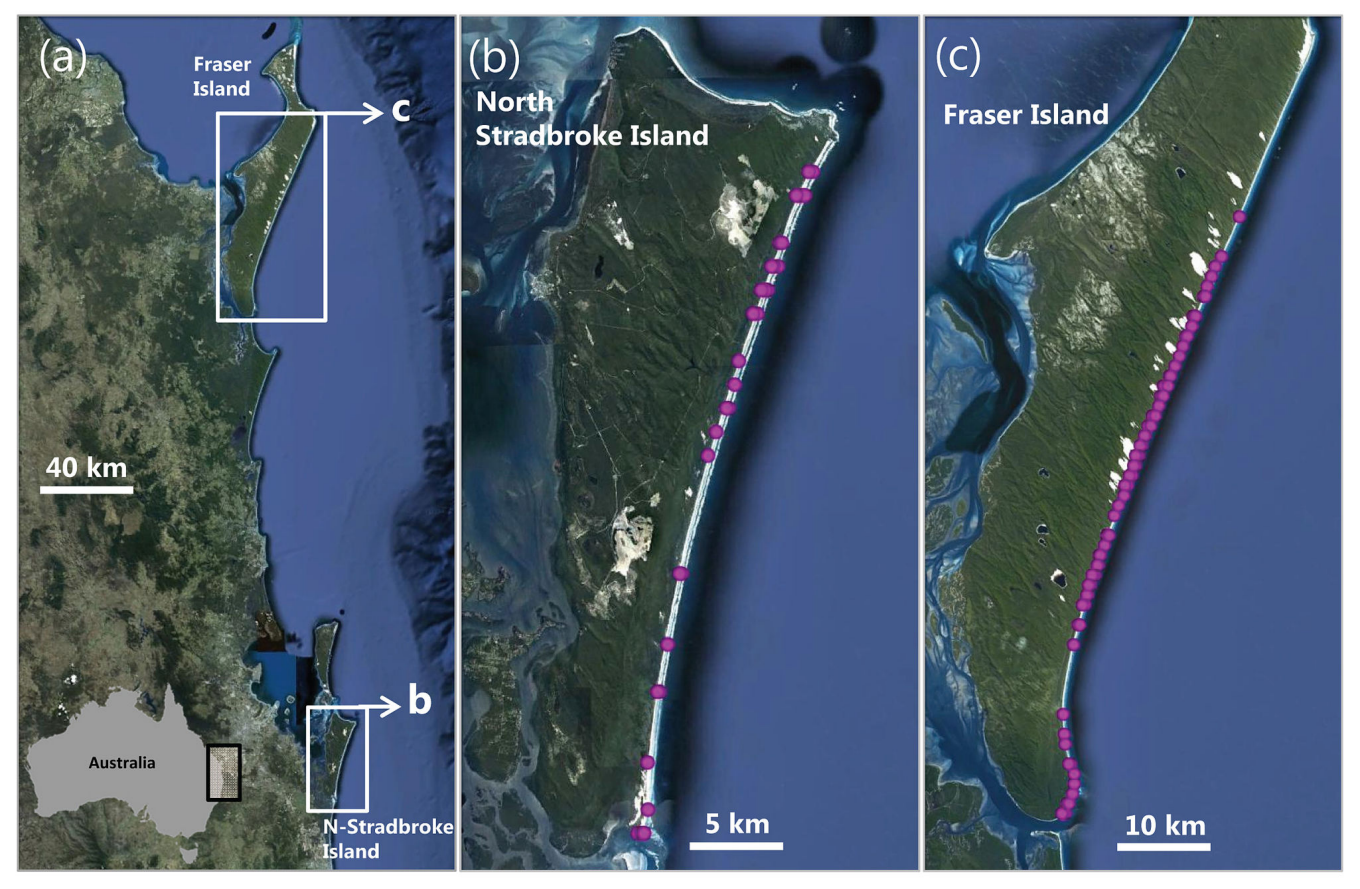
Location of the study sites, Fraser and North Stradbroke Island, in Eastern Australia (a) and positions of focal observations (purple circles) on vehicle-bird interactions on the open-coast beaches of North Stradbroke (b) and Fraser Island (c).

The study was conducted on the ocean-exposed beaches of two barrier islands, North Stradbroke Island and Fraser Island, located off southeast Queensland, Australia (Figure 1). The sites were selected based on four attributes particularly relevant for the study of interactions between vehicles and birds: 1) the eastern shores of both islands feature long stretches of exposed sandy beaches [32], 2) dunes and beaches of both islands are important feeding, roosting, and breeding sites for coastal birds [17,33,34], 3) motorised traffic is very intense on the beaches [35], and 4) this heavy traffic, most of it recreational, causes substantial disturbance to birds [20], resulting in conservation concerns about the impacts of traffic on birds and other wildlife [36,37].

Vehicles on beaches in the region comprise mostly conventional off-road vehicles (‘ORVs’). Other motorised traffic includes tour buses (operated by tourism companies), trucks (service and delivery vehicles), motor bikes and small aircraft that use beaches as a landing strip for scenic flights [20]. We use the term ‘*vehicle*’ to encompass all motorised traffic, ‘*car*’ for 4x4 capable passenger cars (ORVs), and ‘*bus*’ for off-road tourist buses.

### Species selection

Crested terns, *Thalasseus* *bergii* (henceforth ‘terns’) and Australian pied oystercatchers, *Haematopus* *longirostris* (henceforth ‘oystercatchers’) were selected as the model species to examine disturbance to birds because they are abundant on North Stradbroke and Fraser Islands, they occur extensively around sandy shores of Australia, and they have ecological analogues on many sandy shores around the world [38,39] These species encounter vehicles frequently in the study area [20]. While neither species is nationally threatened, factors such as sea-level rise and disturbance are recognised as potential threats [38,39]. Terns are primarily a marine species which roost, and sometimes breed, on sandy shores, while oystercatchers are a sandy-shore obligate species. These species were chosen because they represent two common groups of beach-dwelling bird and, being non-threatened, meant that experiments did not compromise population viability.

### Field methods

Our main objectives were to document how drivers react to the presence of birds on the beach, including driver behaviours that resulted in flushes of individual birds or flocks, and to test the efficacy of setback distances and reduced speeds in lowering disturbance to birds. Consequently, we used an observational phase to document human behaviour and responses of birds, followed by an experimental phase to test whether buffer distances and reduced speed mediate the intensity of disturbance caused by motorised traffic to birds. During the observational phase we estimated distances between vehicles and birds by eye, following an extensive series of ‘observer calibration’ trials: these are described in detail in section 2.7 and in the supporting figures and tables.

Interactions between wildlife and humans can be conceptualised as a sequence of events centred around the birds. First, a ‘*stimulus*’ must be present which is capable of eliciting a response in the species. In the case of recreational traffic on beaches, the stimuli are vehicles of various types and we define the stimulus based on attributes of size and form (e.g. cars, buses, motor bikes) and their behaviour (e.g. speed, whether or not evasive action is evident when birds are present). Second, the stimulus comes into contact with, or proximity to birds, defined here as an ‘*encounter*’, where a vehicle moves along the beach past the birds. Encounters between the stimulus and the wildlife can evoke a ‘*response*’ on the part of the wildlife, defined here as a measurable change to their behaviour. ‘Disturbance’ is the behavioural or physiological change to normal behaviour which occurs as a result of any responses (Weston et al. 2012).

### Observations of vehicle-bird encounters

Two key metrics that define interactions between people and birds on sandy shores are the type of behaviour that humans display towards birds (e.g. the reaction, if any, of drivers when encountering birds whilst driving on the beach), and the type and intensity of the behavioural responses (e.g. the likelihood of birds being flushed or other significant changes to their behaviour). We recorded both these aspects during field observations without interfering with the normal wildlife-human encounters.

Interactions between birds and vehicles were recorded by two observers using binoculars. Observations were made from a vehicle parked on the upper shore near the dunes. The vehicle was at least 100 m from focal birds to minimize observer effects on bird (or driver) behaviour; we checked this by noting whether birds displayed enhanced vigilance, and abandoned further recordings in a few cases where this occurred. Recordings included variables in four classes: i) attributes that define the stimulus (i.e. type of vehicle, speed), ii) the behaviour of drivers once they had encountered birds on the beach (distance between birds and vehicle (cf. Section 2.6), change of speed or direction (i.e. evasive action), iii) the time birds spent engaged in different behaviours in the absence of vehicles (i.e. behavioural time budgets between or before encounters with vehicles), and iv) the intensity of the response resulting from encounters.

Behaviours in the absence of vehicles were categorised according to Schlacher et al. (2013) [20], and measured as the time spent in each category (using the application “IObserver” on an Apple^TM^ Ipad). Changes to behaviour following encounters with vehicles were ranked on an ordinal scale of increasing disturbance intensity: *0 –no change* in behaviour, *1 – vigilance*: at least one individual in the flock alters its behaviour to become vigilant, but no bird flees, either by running or by flying away, *2 – shuffle*: at least one individual in a flock shows a mild escape response by shifting position in the form of a short (< 1 m) run or swift walk; no bird takes flight, 3 -*run*: at least one individual in a flock shows a distinct escape response in the form of a run (> 1 m) away from the stimulus, but no bird takes flight, and 4 – *flight*: at least one individual in a flock takes flight. Our procedure of ranking disturbance response on an ordinal scale resembles methods used in similar bird-human interaction studies [40,41]. We also recorded the following contextual variables: number of individuals in the flock, beach width (m), wind speed (km h^-1^), temperature (°C), and state of the tide (hours since low water); these variables could potentially influence bird behaviour [17,20,32]. Focal observations lasted for 30 minutes or until an encounter with a vehicle, whichever came first. We chose this time cut-off to maximise the probability of obtaining replicate observations between vehicles and birds (the focus of the study) rather than locking up field resources at single locations that did not produce data within a reasonable time period.

Birds were haphazardly ‘sampled’ by making focal observations on birds as we encountered them whilst driving along the beach. To increase the spatial dispersion of focal observations, the along-shore starting position of drives was randomised for each field day. On a given day, birds at a site were sampled only once and the minimum spatial separation to other observation sites was 200 m. For flocks of up to ten individuals we used scan sampling, where each individual in the flock was instantaneously sampled during regular scans [42]. For flocks of more than ten individuals, we haphazardly selected ten birds and applied scan sampling to this subset.

A total of 144 observations of encounters between birds and vehicles were made. To achieve temporal dispersion, surveys were spread out between 01 Oct. 2012 and 18 Jan. 2013. We partitioned total sampling effort to include weekdays (n = 98 observations, 68%) and weekends (n = 46, 32%) at their respective calendar ratio (No. weekdays : No. weekend-days = 1 : 2.5).

### Experimental study

Our second objective was to test the efficacy of setback distances (i.e. spatial buffers between vehicles and birds) and the effects of vehicle speeds on the probability of flushing; this required an experimental approach. Specifically, we wished to measure flushing because this response involves the greatest energy expenditure.

Critical design aspects were the separation distances and the speeds to be tested in the experiments. Distances to be tested were derived from the relationships between separation distance and the intensity of disturbance obtained during the observational phase of the study, tempered by considerations of practicality of implementation of management practices. We used three criteria to identify the distances to be used during the experimental phase of the study:

1) *Distances need to be biologically effective.*
Separation distances must significantly and substantially reduce flushing rates. In encounters with cars, the probability of taking flight declined with greater separation distances for both species. Terns were significantly less likely to be flushed by cars at a separation distance of 25 m or more, and oystercatchers generally did not take to the air if cars passed them at 25 m or wider. Thus, the maximum separation distance adopted in the experiments was set at 25 m.
2) *Distances must be practicable.*
It is unrealistic to recommend (or legislate) separation distances in increments that are difficult to judge by drivers, or to recommend more than one separation distance for different species of birds; the most practicable approach was therefore to use a single ‘minimum distance’ that drivers must maintain between their vehicle and birds.
3) *Distances must be socially acceptable.*
Setback distances must have reasonable public uptake and be realistic. Since 79% of vehicles drove closer than 25 m to birds during our focal observations, a separation distance of 25 m, notwithstanding its biological efficacy, may not be achievable, or would result in very low compliance. Our experiment therefore also included a much less stringent separation distance of 5 m. Minimum approach distances to birds of 5 and 25 m have also been suggested previously [43,44].

Vehicle speeds were selected based on the existing upper speed limit on the open beaches, 80 km h^-1^, contrasted with 30 km h^-1^ which is the limit in pedestrian access zones on the beach (http://www.nprsr.qld.gov.au/parks/fraser/about.html#driving_safely).

Thus, the experimental design consisted of three fixed factors: 1) ‘*Species*’ (tern vs. oystercatcher), 2) ‘*Distance*’ (25 m vs. 5 m), and 3) and ‘*Speed*’ (30 km h^-1^ vs 80 km h^-1^).

The procedure for the field experiment consisted of a fixed sequence of events: i) two observers drove a car (white Land Rover Defender) along the beach until they spotted a flock in the distance; ii) counts of individuals were made with binoculars and the safety of the site assessed (e.g. no approaching vehicles, suitable driving conditions near birds), iii) the vehicle passed the flock at a set distance and speed (i.e. permutations of 25 and 5 m, 80 and 30 km h^-1^ allocated randomly), and iv) the behaviour of the birds was recorded using the intensity of response scale (see above). Sixty experiments were conducted over two days in Feb. 2013 on Fraser Island.

### Distance calibration and diagnostics

Because distance between a stimulus and an organism is often the most critical factor determining the likelihood and intensity of a response by the organism [24], accurate estimation of distances between birds and vehicles is important. Since we estimated this distance by eye in the field (as would be the case if management prescriptions were made), we incorporated extensive protocols to test the accuracy of our estimates and to improve their reliability during both the observational and experimental phase of the study.

During the observational phase of the study, each observer undertook a series of ‘eye calibration’ trials at the start of each day in the field when encounters between birds and vehicles were recorded. Each of these trials consisted of five steps:

1. a model of a bird was placed on the lower beach near the swash (the place where most birds roost and feed);
2. small markers were placed up-shore from this mock bird in the sand at series of distances towards the dunes (1, 2, 5, 10, 15, 20, 25, 30, 35, 40, 45, 50 m);
3. a driver made at least three replicate passes with a vehicle at each of these marked-out distances,
4. observers (independent of drivers) estimated the distance that the vehicle was separated from the mock bird; and
5. the actual distance at which the vehicle passed the mock bird (known to the driver based on the small markers placed on the sand) was compared with the distances estimated by the observer.

The observer was placed on the upper shore near the dunes in the same position from which the actual recordings of bird-vehicles encounters were made. After a number of vehicle passes the observer received feedback on his/her ‘estimates’ to improve the accuracy and repeatability of the distances judged by eye.

The mean distances estimated by the observer did not differ by more than 7% from the actual value of the distance between the mock bird and the vehicle. The maximum residual (i.e. estimated – observed) was 8 m at a test distance of 50 m, representing a deviation of 16% from the actual value. Observers also judged distances with good precision: the standard error of mean estimates made by observers was < 5% of the mean across the full range of distances tested. There was no drift (i.e. directional bias) of distance estimates at increasing values of test distances; the slope of the regression line of estimated vs. actual distances did not differ significantly from a 1:1 line (F_(1,273)_ = 0.63, p = 0.43; Table S1, Figure S1).

For the experimental phase, we trained drivers to maintain fixed distances of 5 and 25 m between their vehicle and a model bird. Drivers drove past a model bird and after each pass received feedback via radio on the actual distance they had achieved. This distance was measured by two investigators after each vehicles pass with a tape measure using the mock bird and tyre imprints in the sand as markers. We repeated this process until all drivers could competently implement a set separation distance between their vehicle and a bird. For both distances tested (i.e. 5 and 25 m), encompassing four drivers in 70 replicate trials, the mean distances that drivers maintained were within 10% of the actual test distance required for all but one driver and distance (max. residual of 18% at 5 m). Half of the mean distances achieved by drivers had a residual better than 5% of the true value (Figure S2). Drivers also achieved the required distances with good precision: standard errors of repeated approaches ranged between 2 and 4% of the mean. Thus, experimental distances were deemed both accurate and reproducible by us.

A central goal of our focal observations was to identify factors (e.g. type of vehicle, speed) that can predict the probability of birds flushing (i.e. encounters that culminate in birds escaping by taking flight). Because variations in the probability of flushing of birds caused by different vehicle types may, theoretically, be influenced by beach width and the state of the tide during which focal observations were conducted (e.g. vehicles approach birds closer when the beach is narrower), we partitioned total variance in beach width and tide state with a Generalized Linear Model (GLM) containing the fixed terms ‘*Species*’ (terns vs. oystercatchers) and ‘*Vehicle Type*’ (cars vs. buses). There were no significant differences in beach width for focal observations on different species (p = 0.48), for different vehicle types (p = 0.76), or for combinations of vehicle type and species (p = 0.48). There were also no systematic differences in the state of the tide during which focal observations were conducted between species, vehicles, or combinations thereof (min. p = 0.43). Thus, systematic bias due to beach driving conditions between treatments is unlikely to have confounded the results.

### Data analyses

The probability of flushing during encounters was the response variable of primary interest, chiefly because of the high energetic costs involved in taking flight as an escape strategy [45].

We modelled the probability of flight (a binary outcome of flying vs. remaining on the ground) using Generalized Linear Models (GLZ) with logit link functions [46]. Saturated GLZ-models contained eight predictor variables: ‘*Species*’, ‘Separation Distance’, ‘*Vehicle Type*’, ‘*Beach Width*’, ‘*Tide*’, ‘*Flock Size*’, and ‘*Air Temperature*’. Model performance was evaluated using the corrected Akaike Information Criterion (AICc) based on all possible combinations of environmental variables used in model building [47,48]. A multi-model inference approach was used to assess the contributions of individual variables, based primarily on their summed Akaike weights [49]; summed AICc weights (w+) provide relative probabilities of variable importance; variables with w+ < 0.3 are likely to be of little or no importance [50].

Variation in response intensity scores was partitioned with Permutational Analysis of Variance, PERMANOVA [51] to account for possible non-normal error structure in the response variable; the crossed design encompassed ‘*Vehicle Type*’ and ‘*Species*’ as fixed factors.

## Results

### Spatial separation between vehicles and birds

Distances that separated birds from vehicles during encounters on beaches ranged from 0 m, where vehicles drove directly at birds, to 55 m. More than one third (39%) of all vehicles passed birds closer than 10 m (n = 56), three-quarters kept a distance of 20 m or less (n = 107), and 90% of vehicles were closer than 30 m to birds (n = 140). Conversely, only 26% of drivers kept a separation distance of at least 25 m (n = 23). A quarter of drivers used less than 40% of the available beach to create a spatial buffer between their vehicles and birds. This indicates that close encounters with birds were not unavoidable due to narrow beaches, but that despite the available space, few drivers utilised a sizeable fraction of this space to lower their effects on birds.

Mean separation distances did not differ significantly between species (p = 0.22), type of vehicle (p = 0.23), or combinations of species and vehicle type (p = 0.49). Buses approached birds closer (terns, 14.71 ± 2.23 m; oystercatchers, 13.60 ± 2.86 m) than cars (terns, 18.68 ± 1.50 m; oystercatchers, 14.65 ± 1.52 m). The incidence of encounters which were 'direct hits' (0 m) or 'near misses' (< 5 m) was 15% for buses and 9% for cars.

### Responses to encounters

Behavioural responses of birds to vehicles were common in both species, despite different habitat use patterns of terns and oystercatchers (Table 1). Terns use sandy beaches exclusively as roosting sites and for social interactions between foraging flights over the surf-zone and marine waters beyond. Oystercatchers forage on beaches, allocating half of their time to foraging (Table 1).

**Table 1:** Proportion of time allocated to different types of behaviour by terns and oystercatchers in periods between encounters with vehicles.

	** *Thalasseus* *bergii* (Crested Tern)**			** *Haematopus* *longirostris* (Australian Pied Oystercatcher)**	
**Behaviour Type**	mean	(se)		mean	(se)
**1. Foraging**	**0.000**	**(0.000)**		**0.587**	**(0.069)**
1.1 Food searching	0.000	(0.000)		0.442	(0.010)
1.2 Probing	0.000	(0.000)		0.063	(0.003)
1.3 Prey Capture	0.000	(0.000)		0.026	(0.002)
1.4 Prey Handling	0.000	(0.000)		0.046	(0.002)
1.5 Swallowing	0.000	(0.000)		0.010	(0.001)
**2. Inter- and Intraspecific Interactions**	**0.001**	**(0.001)**		**0.002**	**(0.000)**
2.1 Antagonistic Behaviour	0.001	(0.001)		0.001	(0.001)
2.2 Courtship	0.000	(0.000)		0.001	(0.001)
**3. Resting, Preening, Miscellaneous**	**0.998**	**(0.088)**		**0.412**	**(0.023)**
3.1 Resting (Roosting)	0.236	(0.009)		0.124	(0.005)
3.2 Maintenance / Preening	0.638	(0.012)		0.053	(0.003)
3.3 Swash Avoidance	0.067	(0.006)		0.109	(0.003)
3.4 Locomotion, General	0.034	(0.001)		0.126	(0.005)
3.5 Thermoregulation	0.023	(0.002)		0.000	(0.000)
**4. ‘Kleptoparasitism’**	**0.000**	**(0.000)**		**0.001**	**(0.001)**

Birds encountered vehicles, on average, within 3 min after focal observations were started (mean time to first encounter; terns: 139.5 ± 16.02 s; oystercatchers: 147.06 ± 21.08). Motorised traffic strongly altered the behaviour and habitat use of birds on the beaches. Of 144 focal observations on bird-vehicle encounters, birds responded in 130 (Table 2). Birds displayed heightened vigilance in 21% of encounters, and escaped from vehicles by walking (14%) and running (17%). Fifty five encounters (38%) ended with flying.

**Table 2:** Frequency of altered behaviour resulting from disturbance by vehicles observed during focal observations of two common bird species on open-coast beaches.

	**0 = none**	**1 = vigilance**	**2 = shuffle**	**3 = run**	**4 = flight/flush**
Crested terns					
	2	10	9	9	47
	(3%)	(13%)	(12%)	(12%)	(61%)
Australian pied oystercatchers					
	12	20	11	16	8
	(18%)	(30%)	(16%)	(24%)	(12%)
Both species					
	14	30	20	25	55
	(10%)	(21%)	(14%)	(17%)	(38%)

Behaviour was scored on a five point ordinal scale of increasing intensity from 0 = none to 4 = escapes by taking flight (see Methods for a full explanation of scoring).

**Figure 2:**
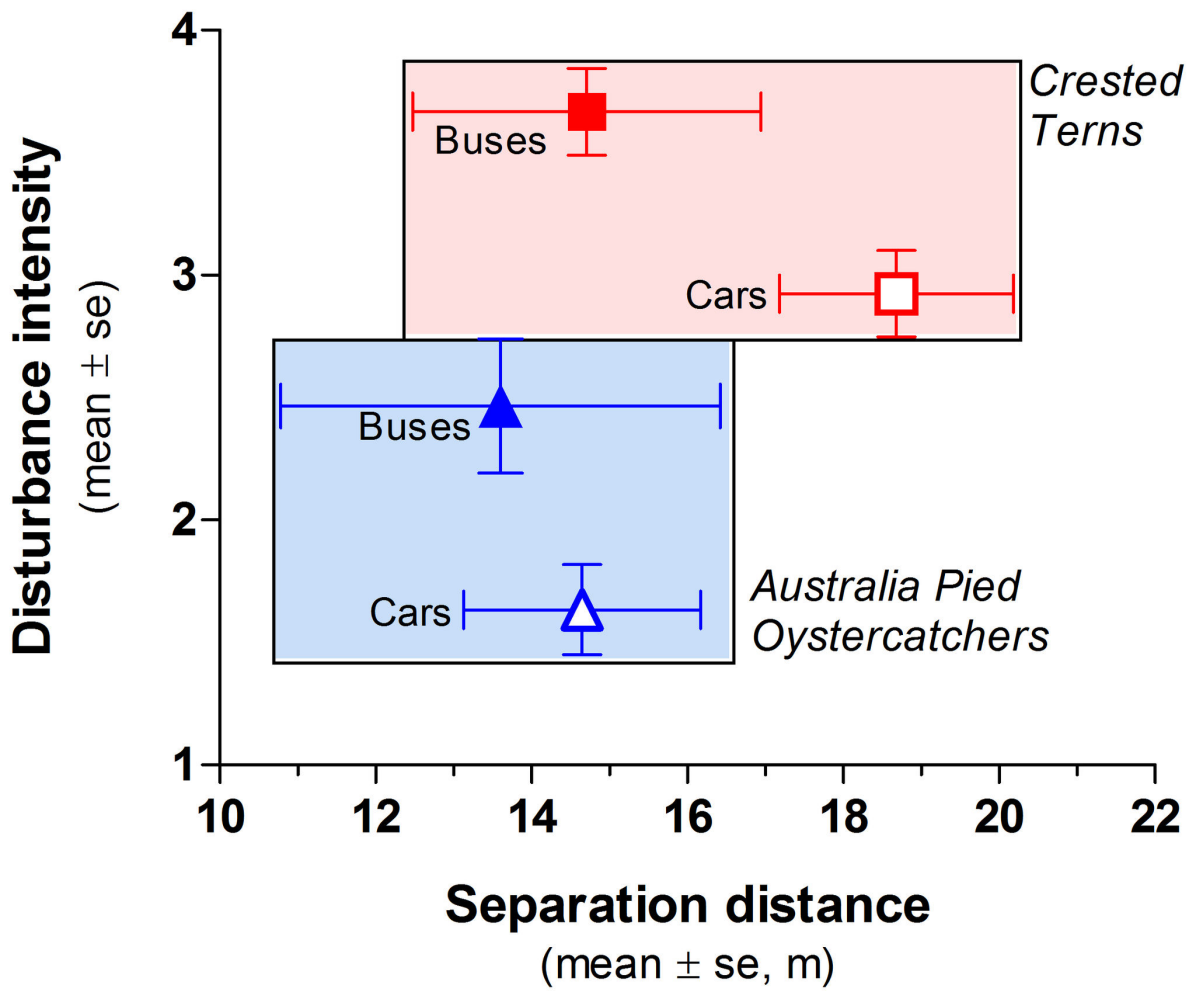
Comparison between buses and cars in terms of the intensity of disturbance-related behaviours shown by birds and the distances separating vehicles from birds for terns and oystercatchers.

Responses differed significantly between species and vehicle types (Table 3, Figure 2). Terns reacted more strongly (1.7 x) than oystercatchers (mean response intensity scores; terns, 3.16 ± 0.14; oystercatchers, 1.82 ± 0.16; Figure 2). Buses evoked measurably larger (1.4 x) response intensities, including more birds responding by fleeing on the wing (mean disturbance scores; cars: 2.29 ± 0.14; buses: 3.21 ± 0.18; Figure 2, Table 3).

**Table 3:** Summary of Permutational Analysis of Variance (PERMANOVA) comparing disturbance scores between species and vehicle types.

**Source**	**df**	**MS**	**Pseudo-F**	**P(perm)**	**No. perms**		**Direction**	**P(perm)**
**Species**	1	42.34	28.39	0.001	994		Terns > Oystercatcher	<0.001
**Vehicle Type**	1	16.92	11.35	0.002	998		Bus > Car	<0.001
Species * Vehicle	1	0.06	0.04	0.856	999			
Residual	140	1.49						

Bold values indicate significant effects.

### Predicting the probability of flushing

Fleeing on the wing (‘flushing’) is energetically the most costly type of escape response, and preventing, or reducing, the incidence of flushing is therefore a conservation priority. To this end, models that can identify factors important in predicting the probability of flushing are of conservation value.

**Figure 3:**
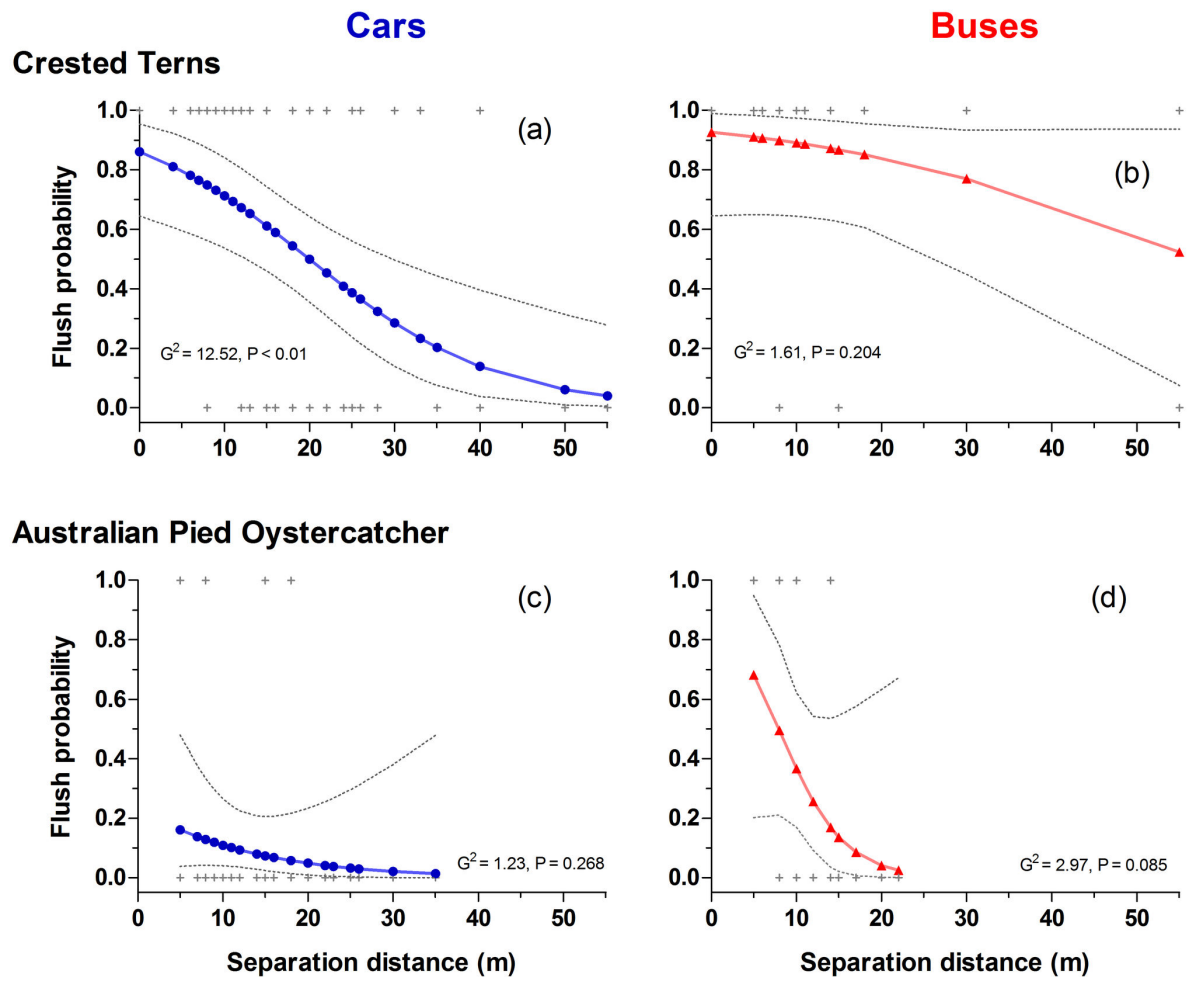
Logistic regressions modelling the probability of flushing (i.e. the probability of birds escaping vehicles by taking flight) of terns (a and b) and oystercatchers (c and d) in relation to distance between birds and cars (left column), and birds and buses (right column). Solid lines connect predicted probabilities at observed distances; dotted lines are 95% confidence limits of model predictions, and crosses are observed flight responses.

The best overall model predicting the flush rate of birds contained four predictors: species, vehicle type, distance, and beach width (Table 4). Larger separation distances significantly decreased the probability of birds flushing (odds ratio = 0.91; Figure 3, Table S2), whereas birds were very marginally more likely to take flight when the beach was wider (OR = 1.01). Response patterns differed strongly between species, with terns much more likely to take flight (OR = 5.76). Buses were twice as likely to flush birds (OR = 2.00) than were cars (Table S2).

**Table 4:** Summary of Generalized Linear Models (GLZ) analysing the probability of birds being flushed in encounters with vehicles (i.e. a binary outcome of flight or no flight) as a function of nine predictors.

No. Variables	AICc	ΔAIC	w_i_	Species	Distance	Vehicle Type	Beach Width	Flock Size	Speed	Tide	Spp *Dist.	Temp.	Wind
1	163.46	25.23	<0.001	x									
2	146.89	8.60	0.001	x	x								
3	139.29	0.92	0.046	x	x	x							
*4	138.49	0.00	0.069	x	x	x	x						
5	140.16	1.52	0.030	x	x	x	x	x					
6	141.16	2.35	0.018	x	x	x	x	x	x				
7	143.10	4.07	0.007	x	x	x	x	x	x	x			
8	145.07	5.80	0.003	x	x	x	x	x	x	x	x		
9	147.12	7.58	0.001	x	x	x	x	x	x	x	x	x	
10	149.43	9.58	<0.001	x	x	x	x	x	x	x	x	x	x

The best model (based on AICc) for each number of predictor variables is shown, with * denoting the best overall model, and ‘x’ denoting inclusion of a variable in a model for a given number of predictors.

Summed variable weights provide relative probabilities of variable importance, and this analysis shows that three variables were clearly most influential in our models predicting the probability of flushing of birds: species, the type of vehicle (bus or car), and the separation distance between vehicles and birds (Table 5). Beach width had a moderate, but non-significant (p= 0.09), influence on flushing. All other variables included in our models, were inconsequential in explaining the probability of flushing; unimportant variables included attributes of the habitat (state of the tide), weather (temperature, wind speed), a biological trait (flock size), and a property of the stimulus itself (vehicle speed).

**Table 5:** Contributions of variables to GLZ models used to predict the probability of birds being flushed by vehicles.

Variable	Best Model	Prop. models with Δi ≤ 4	w+(j)	Wald Statistics	P
**Species**	*	1.00	1.00	28.52	0.000
**Separation Distance**	*	1.00	1.00	13.82	0.000
**Vehicle Type (bus, car)**	*	0.97	0.90	5.64	0.018
Beach Width	*	0.65	0.60	2.92	0.087
Vehicle Speed		0.35	0.41	1.23	0.268
Tide		0.24	0.29	0.34	0.562
Flock Size		0.24	0.28	0.40	0.528
Temperature		0.24	0.27	0.20	0.654
Species * Distance		0.24	0.27	0.26	0.612
Wind Speed		0.24	0.25	0.00	0.969

Variable contributions are assessed in a multi-model inference approach using cumulative weights (w+(j)), and the proportion of models with Δi <=4 in which a variable was included as the primary criteria. Variables in bold have coefficients with P < 0.05 in marginal tests.

### Experimental tests on the efficacy of setback distances and speeds

The purpose of the experiments was to test combinations of speed and setback distances which may underpin management recommendations. We recorded sizeable (-44%) and significant (p = 0.004) reductions in mean response intensity scores when drivers slowed to 30 km h^-1^ and when they created a buffer between their car and birds of 25 m (Table 6). Increasing the separation distance from 5 to 25 m halved the response intensity score from 4 (i.e. flush) to 2 (i.e. shuffle; Table 6). Increasing the separation distance was generally more important in reducing responses than were changes to vehicle speed. Substantial declines in response intensity scores at 25 m were recorded at both speeds, whereas lowering the speed only produced a sizeable effect when cars were more distant from the birds (Table 6).

**Table 6:** Comparison of disturbance intensity in terns during experimental encounters with vehicles at two speeds (30 vs. 80 km h^-1^) and two separation distances (5 vs. 25 m).

	**Speed: 30 km h^-1^**					**Speed: 80 km h^-1^**						
**Separation** **Distance**	**n**	**mean**	**(se)**	**median**		**n**	**mean**	**(se)**	**median**		**Contrast**	**P**
5 m	17	3.4	(0.35)	4		12	3.7	(0.26)	4		30 vs 80 kmh^-1^	0.578
25 m	18	1.9	(0.28)	2		13	2.8	(0.39)	4		30 vs 80 kmh^-1^	0.057
	5 vs 25 m	P = 0.004				5 vs 25 m	P = 0.119					

The behavioural response of terns was scored on a five point ordinal scale of increasing disturbance intensity (0 = none, 1 = vigilance, 2 = shuffle, 3 = run, 4 = flight/flush, cf. methods for a full explanation); tabulated values represent statistics of these scores. P-values refer to pairwise contrasts (rows: distance effects; column: speed effects) of means in Permutational Analysis of Variance (PERMANOVA).

**Figure 4:**
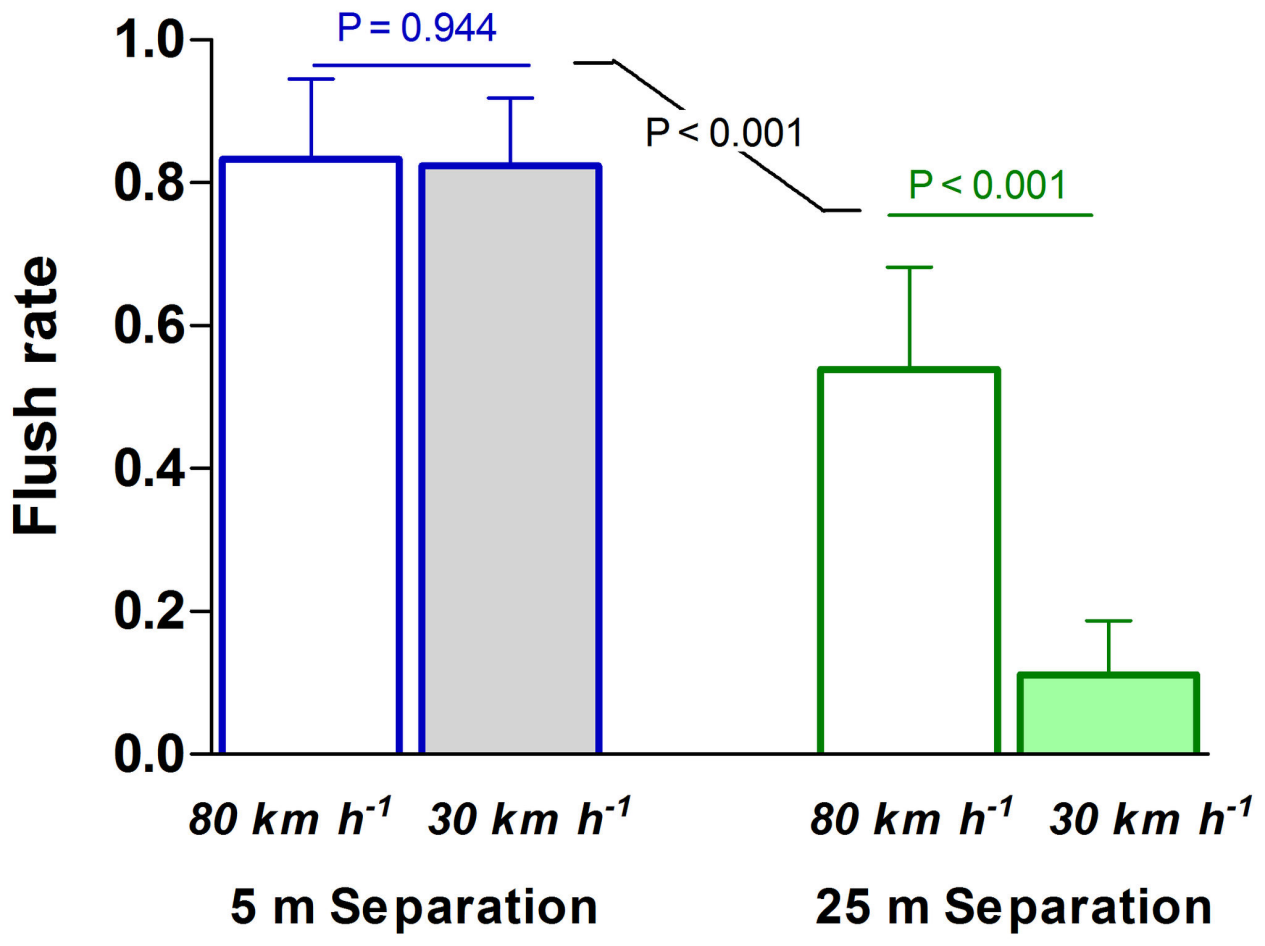
Effects of separation distance and vehicle speed on flush rate in crested terns during experimental encounters with vehicles on open-coast beaches.

Terns flushed at rates which permitted further analysis of the probability of flushing. Separation distance had a large and significant effect on probability of flushing terns in the experiments (GLZ distance term; G^2^ = 16.90, P < 0.001). At a separation distance of 5 m, four out of five passes resulted in terns being flushed (Figure 4). When distances were increased to 25 m, the probability of terns flushing declined significantly to half or less (Figure 4). Overall, terns were 72% more likely to get flushed when cars passed them at 5 m (Figure 4).

Vehicle speed influenced flushing rates less than did distance (GLZ speed term; G^2^ = 2.98, P = 0.092) in the experiments, being to some degree also contingent upon separation distance (GLZ dist. * speed term; G^2^ = 2.46, P = 0.116). When vehicles passed birds at 30 km h^-1^ and at a separation distance of 25 m, only a single experimental encounter culminated in a flush (Figure 4). Conversely, vehicles travelling at the legal speed limit of 80 km h^-1^ flushed 54% of birds even when a separation distance of 25 m was maintained. Speed had no significant effect (P = 0.945) on the proportion of encounters evoking flushes when vehicles passed birds closely at 5 m (Figure 4). Overall, our model predicting probabilities of flushing using vehicle speed and distance as predictors correctly classified 78% of responses.

## Discussion

Vehicles using the beaches caused frequent and substantial disturbance effects to wildlife, with many drivers approaching birds close and at high speeds to force escapes on the wing. Classic models of human disturbance to birds posit that vehicles evoke responses from wildlife that are less frequent or intense than those evoked by pedestrians [24,52]. These models do, however, rarely consider the speed or distance covered by vehicles, the large number of vehicles in some parks, and the rate at which they encounter wildlife, but see 20,23 - all factors that contribute to the considerable disturbance effects recorded here.

Effective setback distances are considered species- and situation specific [27]. The overall response distances we report here are within the broader range, 14-126 m, of flight initiation distances (FIDs) reported for shorebirds [24]. Very few studies on effective buffers for birds against anthropogenic stimuli test vehicles or are conducted on beaches [24]. Of the studies that measured the response of birds to vehicles [53,54], none report responses of terns or oystercatchers. In our study, about half of all encounters between terns and vehicles resulted in flights at separation distances of ca. 20 m (Figure 3). This is very broadly comparable to distances reported for terns when approached by walkers, i.e. 17 m [24]. This similarity suggests that terns may not always discriminate between vehicles and walkers in terms of escape responses. Alternatively, terns could have altered their response distances via learning or perhaps local selection - controlled studies are required to test this. On the other hand, the response distances of pied oystercatchers recorded by us (i.e. < 10% of encounters resulting in flight at 20 m; Figure 3) appear shorter than those reported elsewhere, being 39-83 m when approached by walkers [24]. It is commonly suggested that vehicles evoke bird responses at shorter distances than walkers although this has been virtually unstudied, but see 55. This possible difference warrants further investigation using properly controlled comparisons, especially because people will often walk from vehicles and have the potential to disturb birds further when doing so.

### Attributes of disturbance responses and separation distances

One of the key findings of our study is that terns are more sensitive to vehicles than oystercatchers. Oystercatchers are waders and can move rapidly on foot. By contrast, terns have webbed feet and cannot move rapidly on the ground, being forced to take flight for rapid escapes. Terns use the lower beach for roosting and social interactions only, feeding in the surf zone and beyond. By contrast, habitat use in oystercatchers is more diverse, the species using beaches as feeding, roosting and breeding sites [32]. Oystercatchers use parts of the beach which is not traversed by the same number of vehicles (i.e. the wet swash zone near surf) and feeding patches in this zone confer considerable value to birds, making oystercatchers less likely to leave. Regardless of the cause of species differences, the fact that they exist emphasises the need tailor management solutions to accommodate species and situations where most bird responses occur.

One of the salient findings of our study, and one which has management and conservation implications, is that buses are significantly more disruptive to birds than passenger cars. The substantially larger effect of buses results most likely from a combination of driver behaviour and the characteristics of the stimulus itself. Bus drivers tended to approach birds closer than drivers of passenger cars, and birds reacted more strongly to buses, escaping on the wing in the majority of encounters. Birds perceive humans, and, presumably, vehicles, as potential predators, eliciting behavioural responses designed to lower the risk of predation [56]. It is therefore possible that because of their larger size, often greater noise, and consistent non-evasive behaviour (i.e. bus drivers never altered course or reduced speed to avoid birds), buses represent a more threatening stimulus and hence elicit stronger responses. Traffic noise negatively affects wildlife [57], and other anthropogenic noise stimuli are known to elicit behavioural disturbance in birds, such as barking dogs [58]. Although we did not measure noise directly, buses are generally noisier than most cars, and so may provide more threatening auditory cues to birds.

Few studies have disentangled the effect of stimulus speed from stimulus type [27]. We did not find a significant effect of speed on the probability of flushing, and our experiments suggest that speed is less important than distance in eliciting escape on the wing. Speed is, however, a significant predictor of roadkills in vertebrates [5], and fatal collisions occur between birds and vehicles on beaches [20]. Thus, it would be expected that speed would mediate risk to birds, and so would be used by birds to mediate their responses to vehicles [56]. Birds may be unable to judge speeds, especially for extremely rapid approaches (e.g., 80  kmh^-1^), when the time lag between detection of the stimulus and decision to fly may be short. Thus, vehicles may be so dangerous, vehicle speed so variable (i.e. unpredictable), and the risk associated with delayed response so high, that birds have generalised their response to vehicles regardless of the speed at which they occur. Thus, while reductions in speed *per se* will, based on our experimental data, make only a small contribution to reduce disturbance levels to birds, speed limits are presumably very important in lowering avian fatalities on beaches and hence should form part of a broader conservation toolbox.

Birds experiencing repeated encounters with predictable, benign human stimuli may respond less over time, suggesting habituation [59]. Conversely, animals that are frequently disturbed by dangerous stimuli, especially when they are unpredictable, can become sensitized to react more strongly [40]. Vehicles are a frequent stimulus at the study site (i.e. > 250 000 vehicles per year [15], but are not a benign stimulus, because a lack of response or a response not made adequately or quickly enough, causes mortality [20]. Vehicles are also unpredictable, in terms of speed and whether drivers adopt evasive behaviour or not, and this unpredictability may explain the intense responses of birds. From a management perspective, plasticity on the part of the birds is not evident, so encounters between vehicles and birds require management interventions.

### Management and Conservation Implications

A large and growing body of research demonstrates that motorised recreation has numerous and widespread detrimental effects at multiple levels of ecological organisation [10,13,60,61]. The intensity of environmental concerns arising from beach traffic is such that it calls for the design and implementation of management interventions that reduce environmental harm and minimize conflict between motorised and non-motorised recreationists [62]. At the core of interventions that best achieve these goals are measures that separate the threat (vehicles) from sensitive ecological or human targets (e.g. habitats, wildlife), by creating spatial exclusion zones in the form of permanent or temporary beach closures [63,64].

Despite evidence for rapid and significant conservation returns for coastal birds generated from removing vehicles [31,64–67], almost all of the beaches at our study sites remain open to vehicles, both inside and outside of public lands designated explicitly for conservation. Because the core management tool (i.e. spatial refuges) is not available to reduce human-wildlife conflicts in these conservation areas, alternatives need to be scoped. One alternative suggested by the management authorities is that drivers adopt behaviours less harmful to wildlife when encountering birds on beaches (i.e. ‘codes of conduct’). Such codes often feature low compliance and their efficacy in reliably changing behaviour of tourists or recreationists is questionable [68]. Vehicle owners are not discouraged from driving on beaches.

Current behaviours underlying recreational activities based on motor vehicles require fundamental changes to reduce their currently large ecological impacts, on birds and other features of the beach-dune environment [10,35,69]. While the most, and arguably only, effective solution is to drastically reduce traffic volumes or establish traffic-free areas on beaches, changing driver behaviour during encounters with birds can, hypothetically, be an interim measure to lower impacts on wildlife [70].

We showed that separation distances of at least 25 m between birds and vehicles, combined with cars travelling 30 km h^-1^ or slower, measurably reduce disturbance to one common species of coastal bird, crested terns. By contrast, the great majority of drivers currently approach birds much closer and at much greater speeds. This juxtaposition of a desired target behaviour (i.e. 25 m buffer and slow speed) with the prevailing behaviour of beach users (i.e. close encounters at higher speeds) poses a formidable management challenge.

Changing behaviours towards better environmental behaviour is complex. Monroe [71] suggest that, in the context of the theory of planned behaviour, interventions should provide information about the consequences of the behaviour, the ease at which change can be adopted, its effectiveness, and its social acceptability. To encourage appropriate behaviour during encounters with birds, three aspects of the target behaviour can be communicated readily to beach users: 1) the consequences of the current behaviour (i.e. significant disruption of wildlife), 2) the efficacy of the action (i.e. lower probability of flushing), and 3) the ease of implementation (i.e. beaches are generally wide enough to create 25 m buffers between birds and vehicles).

Education is frequently suggested as a tool to promote better environmental stewardship and ecologically more benign behaviour, but it is often unclear to which extent this translates into tangible conservation benefits [72–74]. The link between awareness and positive outcomes for wildlife is tenuous in the case of motorised traffic and birds. Beach users in the region consider driving of vehicles on the beach to cause ‘*extreme*’ levels of disturbance to shorebirds [75], yet the practice continues unbridled [20]. Arguably, insufficient education about the numerous negative ecological effects caused by vehicles may, paradoxically, lead to negative conservation outcomes. This situation may arise for motorised beach recreationists who undertake commercial off-road training courses that may engender some sense of environmental literacy, but do not strongly discourage vehicle owners from driving on the beach.

Despite the popularity of beach driving in some sectors of society, the nature of the activity itself, and its numerous and widespread environmental consequences, can engender conflicts between motorised and non-motorised users [76]. These conflicts can be particularly insidious when non-motorised users are subjected to disproportionally greater impacts caused by motorised recreationists, including direct disturbance of non-motorised beach users, safety hazards, diminished opportunities for wildlife viewing, and loss of the wilderness character and aesthetic values of the area [62]. Only negligible opportunities currently exist for non-motorised recreational users on the beaches studied by us, particularly on Fraser Island; this lack of social equity runs counter to the generally accepted mandate of public lands to adequately provide for multiple uses [3].

Accreditation schemes regarding ecologically sensitive tourism were entirely ineffective in this study: drivers of buses operating as accredited ‘eco tours’ disturbed birds more than did the ‘unaccredited’ drivers of non-commercial cars which behaved more eco-friendly. All bus traffic impacting shorebirds is generated by commercial tour operators transporting visitors around the island - a tourism activity that frequently employs ‘green’ marketing credentials (e.g. ‘eco’, ‘nature-based’, etc.) or uses accreditation which purports to safeguard natural assets including wildlife. The broader question of whether tourism can be a sustainable activity in national parks is a multi-faceted and complex one, including environmental impacts caused by ‘ecotourism’ [1,77,78]. In the current situation there is a clear and urgent need to correct environmentally harmful practices within the industry (e.g., lack of evasive actions by bus drivers, frequent impacts on birds) to decrease tourism’s ecological impacts on beaches and dunes.

## Conclusions

1. Vehicles driven on sandy shores frequently and intensely disturb birds on open-coast beaches.
2. The intensity and probability of a disturbance response is a function of the distance that separates birds from vehicles. Thus, sufficiently wide setback distances are expected to reduce impacts by vehicles.
3. Our experimental test of practical setbacks (and speeds) showed that they will reduce, but not eliminate, disturbance for birds. Setback distances therefore *can* make a contribution to reducing impacts on wildlife and *can* be an effective conservation tool, provided that they are carefully planned and tested.
4. In this case of beach driving, setback distances will, however, never be as effective as the creation of spatial refuges from motorised recreationists (i.e. permanent or temporary beach closures), and are thus only a complementary and temporary measures that cannot replace beach closures.
5. The question as to how much disturbance is tolerable, or how much vehicle-based mortality is required to compromise population persistence, remains unknown.
6. Paradoxically, the ‘eco-friendly’ tourist industry operating buses caused greater disturbance to birds, suggesting that organised tourism – notwithstanding its ‘eco accreditation - does not necessarily engender better outcomes for wildlife. This unexpected result calls for improved industry commitment to conservation.
7. Any solution that has tangible conservation outcomes with regards to the issue of bird- vehicle interactions on beaches will require changes to the status quo and hence may engender controversy.

## Supporting Information

Table S1 **Summary statistics of estimates made by observers who judged the distance separating birds from vehicles.** Each trial involved a model bird and a number of set distances (1 to 50 metres) repeatedly tested at the start of each field observation day. Observers were near the dunes, ca. 100 m distant from a model bird that other members of the study team (not the observers) passed in a vehicle at distances marked out in the sand; these markers were small enough not be seen by the observer.
(DOCX)

Table S2 Summary statistics of logistic regressions, modelling the probability of birds being flushed (i.e. escaping vehicles by taking flight) in relation to separation distance between birds and vehicles.
(DOCX)

Figure S1 Relationship between estimated (by observers in the field) and actual (measured with markers from a model bird) distances separating birds from vehicles on beaches (n = 270 trials).
(TIF)

Figure S2 **Evaluation of driver performance in experiments requiring the maintenance of fixed separation distances between birds and a vehicle.** Distances tested were 5 m and 25 m. Shaded bands encompass two standard deviations around the mean. Deviance is expressed as the percentage difference between the mean distance maintained by a driver and the required test distance.
(TIF)
