# Supplementary material for: Setback Distances as a Conservation Tool in Wildlife-Human Interactions: Testing Their Efficacy for Birds Affected by Vehicles on Open-Coast Sandy Beaches
Source: PLoS One. 2013 Sep 5;8(9):e71200. doi: 10.1371/journal.pone.0071200 (PMC3764142; doi:10.1371/journal.pone.0071200)
Supplement: Table S1 — Summary statistics of estimates made by observers who judged the distance separating birds from vehicles. Each trial involved a model bird and a number of set distances (1 to 50 metres) repeatedly tested at the start of each field observation day. Observers were near the dunes, ca. 100 m distant from a model bird that other members of the study team (not the observers) passed in a vehicle at distances marked out in the sand; these markers were small enough not be seen by the observer. (DOCX) [file pone.0071200.s001.docx]

**Table S1**Summary statistics of estimates made by observers who judged the distance separating birds from vehicles. Each trial involved a model bird and a number of set distances (1 to 50 metres) repeatedly tested at the start of each field observation day. Observers were near the dunes, ca. 100 m distant from a model bird that other members of the study team (not the observers) passed in a vehicle at distances marked out in the sand; these markers were small enough not be seen by the observer.

| **Test Distance** | **No. trials (n)** | **Mean estimated distance (m)** | **SE, estimated** | **SE/mean** | **Residual mean (m)** | **Residual max (m)** |
| --- | --- | --- | --- | --- | --- | --- |
| 1 | 20 | 1.00 | 0.00 | - | 0.0 | 0.0 |
| 2 | 20 | 2.10 | 0.07 | 0.033 | 0.1 | 1.0 |
| 5 | 18 | 5.22 | 0.29 | 0.055 | 0.4 | 2.0 |
| 10 | 19 | 10.74 | 0.44 | 0.041 | 1.4 | 3.0 |
| 15 | 20 | 15.00 | 0.30 | 0.020 | 1.1 | 3.0 |
| 20 | 22 | 21.14 | 0.32 | 0.015 | 1.7 | 4.0 |
| 25 | 23 | 24.83 | 0.36 | 0.014 | 1.4 | 4.0 |
| 30 | 25 | 31.44 | 0.28 | 0.009 | 1.7 | 4.0 |
| 35 | 25 | 35.36 | 0.36 | 0.010 | 1.6 | 4.0 |
| 40 | 25 | 41.48 | 0.36 | 0.009 | 1.9 | 5.0 |
| 45 | 27 | 45.37 | 0.39 | 0.009 | 1.6 | 6.0 |
| 50 | 26 | 52.04 | 0.51 | 0.010 | 2.9 | 8.0 |
