# Supplementary material for: Setback Distances as a Conservation Tool in Wildlife-Human Interactions: Testing Their Efficacy for Birds Affected by Vehicles on Open-Coast Sandy Beaches
Source: PLoS One. 2013 Sep 5;8(9):e71200. doi: 10.1371/journal.pone.0071200 (PMC3764142; doi:10.1371/journal.pone.0071200)
Supplement: Table S2 — Summary statistics of logistic regressions, modelling the probability of birds being flushed (i.e. escaping vehicles by taking flight) in relation to separation distance between birds and vehicles. (DOCX) [file pone.0071200.s002.docx]

**Table S2**Summary statistics of logistic regressions modelling the probability of birds being flushed (i.e. escaping vehicles by taking flight) in relation to separation distance between birds and vehicles.

|  |  | ***Thalasseus bergii* (Crested Tern)** | | | | |  | ***Haematopus longirostris*  (Australian Pied Oystercatcher)** | | | | |
| --- | --- | --- | --- | --- | --- | --- | --- | --- | --- | --- | --- | --- |
|  |  | **Cars** | |  | **Buses** | |  | **Cars** | |  | **Buses** | |
| Model Parameters |  | mean | (se) |  | mean | (se) |  | mean | (se) |  | mean | (se) |
| Intercept |  | 1.818 | (0.624) |  | 2.547 | (0.994) |  | -1.214 | (1.179) |  | 2.066 | (1.948) |
| Beta |  | -0.091 | (0.030) |  | -0.045 | (0.035) |  | -0.088 | (0.090) |  | -0.261 | (0.184) |
|  |  |  |  |  |  |  |  |  |  |  |  |  |
| Odds ratio |  | 0.91 |  |  | 0.96 |  |  | 0.92 |  |  | 0.77 |  |
|  |  |  |  |  |  |  |  |  |  |  |  |  |
| G^2^ |  | 12.53 |  |  | 1.61 |  |  | 1.23 |  |  | 2.97 |  |
| P |  | <0.001 |  |  | 0.204 |  |  | 0.268 |  |  | 0.085 |  |
|  |  |  |  |  |  |  |  |  |  |  |  |  |
| Hosmer Lemeshow |  | 7.02 |  |  | 5.58 |  |  | 4.78 |  |  | 4.44 |  |
| P |  | 0.43 |  |  | 0.35 |  |  | 0.69 |  |  | 0.73 |  |
|  |  |  |  |  |  |  |  |  |  |  |  |  |
| Classification |  |  |  |  |  |  |  |  |  |  |  |  |
| No. cases |  | 57 |  |  | 20 |  |  | 48 |  |  | 19 |  |
| No. correctly classified |  | 39 | (68%) |  | 17 | (85%) |  | 44 | (92%) |  | 14 | 74% |
